# Computational *de novo* discovery of distinguishing genes for biological processes and cell types in complex tissues

**DOI:** 10.1371/journal.pone.0193067

**Published:** 2018-03-01

**Authors:** Lee A. Newberg, Xiaowei Chen, Chinnappa D. Kodira, Maria I. Zavodszky

**Affiliations:** Biosciences, General Electric Global Research Center, Niskayuna, NY, United States of America; Universita degli Studi di Torino, ITALY

## Abstract

Bulk tissue samples examined by gene expression studies are usually heterogeneous. The data gained from these samples display the confounding patterns of mixtures consisting of multiple cell types or similar cell types in various functional states, which hinders the elucidation of the molecular mechanisms underlying complex biological phenomena. A realistic approach to compensate for the limitations of experimentally separating homogenous cell populations from mixed tissues is to computationally identify cell-type specific patterns from bulk, heterogeneous measurements. We designed the CellDistinguisher algorithm to analyze the gene expression data of mixed samples, identifying genes that best distinguish biological processes and cell types. Coupled with a deconvolution algorithm that takes cell type specific gene lists as input, we show that CellDistinguisher performs as well as partial deconvolution algorithms in predicting cell type composition without the need for prior knowledge of cell type signatures. This approach is also better in predicting cell type signatures than the one-step traditional complete deconvolution methods. To illustrate its wide applicability, the algorithm was tested on multiple publicly available data sets. In each case, CellDistinguisher identified genes reflecting biological processes typical for the tissues and development stages of interest and estimated the sample compositions accurately.

## Introduction

It has become a common practice to obtain bulk tissue samples from patients and perform gene or protein expression analysis on them for diagnostic purposes. However, these samples are most often composed of multiple cell types, contain cells inadvertently captured from adjacent tissues, or are composed of cells dominated by different biological processes. An accurate estimate of the tissue composition and the gene expression levels of the cellular subtypes can have diagnostic value in terms of differential diagnosis and staging of the disease. It can also shed light into the mechanism of disease progression. Experimentally separating the various cells and then analyzing their gene expression signatures is technically challenging, time consuming, and costly. As an alternative, computational approaches of deconvolution have been designed to identify the different cell types from mixed samples using gene expression data from RNA sequencing or microarrays, or using protein expression data [1–4]. Generically, we will refer to gene expression data throughout this article as the default application.

Partial deconvolution algorithms take gene signatures of pure or sorted cell types as an input to determine the fractions of different cell types in mixed samples. Most of them can also do the opposite: taking the tissue composition as input, they provide the gene signatures characteristic to the component cell types. This is a relatively easy mathematical problem limited mainly by the experimental errors measuring the required input. Examples of such partial deconvolution methods are lsfit and cs-lsfit [5] which use least-squares fitting, or the quadratic programming approaches used in qprog [6] and DeconRNASeq [7]. The drawback of these methods is that they require hard-to-obtain information on either the sample compositions or high-quality pure cell-type signatures.

Complete deconvolution is a more challenging task. For this, no information is available aside from the expression values in multiple heterogeneous tissue samples and an estimate of the number of individual cell types. Deconf is an example of this approach using non-negative matrix decomposition [8]. When testing deconf on artificially mixed test data, we found that its performance was limited even on noise-free data, and that the performance deteriorated further as increasing amounts of noise were added to mimic the real data; see the Results section.

Unsupervised, complete deconvolution methods work better if prior knowledge is included in the form of marker gene sets [9]. More generally, expression of distinguisher (marker) genes highlight the extent of known and novel biological processes exhibited in heterogeneous samples. Unfortunately, such data are not necessarily available for the cell types and conditions of interest. This was our motivation for developing CellDistinguisher to computationally identify distinguishers in bulk tissues. The underlying algorithm is inspired by a similar topic modeling problem and its performance on data sets of increasing complexity is described below. We compared our approach to existing and published methods incorporated into the R package CellMix [10, 11], including lsfit and cs-lsfit [5], qprog [6], ssKL and ssFrobenius [9] for partial deconvolution, and deconf [8] for complete deconvolution.

While working on refining CellDistinguisher and identifying good test cases, the work of Wang and colleagues was published describing an approach similar to ours in its design. The unsupervised convex analysis of mixtures (CAM) approach is intended to identify markers of subpopulations and the composition of complex biological samples without the need of prior information [12]. We outline the algorithmic differences between CAM and CellDistinguisher in the Methods section of the S1 File, and show (in the Results section of S1 File) that our predictions on three datasets analyzed by both groups, are equally close to the ground truth.

The use of CellDistinguisher is not limited to identifying distinguishers or deconvolving mixtures of cell types. Given a series of samples representing relatively homogenous cell types at different stages of a biological process, we demonstrate that the algorithm can identify representative genes of the various stages. In our examples, these are stages in B-cell development and phases of the mitotic cell cycle in yeast. Besides the representative gene sets, an estimate of the percentage of cells that show the expected stage-specific behavior can also be inferred for every sample.

CellDistinguisher can be used in bioprocessing and cell therapy manufacturing to identify genes that represent biological processes that differentiate successfully engineered cells from unproductive cells and to determine the relative purity of the target cell types in the production line. Measurements of expression of these distinguishing genes can be used for quality assurance and control. Similarly, CellDistinguisher can be used to infer relative ratios of tumor-stroma cells and immune cell types in cancer tissues.

## Material and methods

### Relation to topic modeling

The algorithm we developed exploits the similarity between the computer science topic modeling problem [13] and the present problem, that of identifying genes whose expression is more-or-less unique to a specific biological process or cell type. In topic modeling, the relevant entities are documents, topics, and words. Each document includes one or more topics, in proportions that are to be discovered. Each topic has a to-be-discovered probability distribution of the words used in discourses on the topic. A word is distinguishing for a topic if it is more-or-less exclusive to that topic. Distinguishing words are helpful in several ways including that a census of distinguishing words in a document gives much information about the proportions of the topics in that document.

In the present problem, the parallels to documents, topics, and words are biological samples, biological processes, and genes. By biological processes we mean processes carried out by a cell, including but not limited to those that are specific to only certain cell types or cell subtypes. Each biological sample is typically composed of multiple different cell types exhibiting many biological processes. Each biological process is typified by specific expression levels of a subset of genes. Alternatively, depending on the desired granularity, biological processes can be characterized by expression values at the exon, microarray probe or other structure at the sub-gene level. A gene is distinguishing if it is more-or-less exclusive to that biological process.

### Mathematical recognition of distinguishing genes

The approach begins with the observation that a biological sample that contains a mixture of multiple biological processes will have a gene expression signature that is similar enough to a corresponding mixture of the expression signatures typical of the biological processes. There is a corresponding statement in terms of the matrix of gene-gene conditional expression values, where the (*i*,*j*)-th matrix entry is ${\bar{Q}}_{i,j}=\mathrm{Pr}\left[f_{2}=j|f_{1}=i\right]$, the probability that a second sequence fragment taken randomly from a randomly selected biological sample will belong to gene *j* if the first sequence fragment taken randomly from that biological sample belongs to gene *i*. In this way, each gene *i* is associated with a vector of conditional expression numbers $\left({\bar{Q}}_{i1},{\bar{Q}}_{i2},\ldots \right)$ that has as many entries as there are genes with expression measurements. The vector of conditional expression information for an arbitrary gene will be similar enough to a linear combination of the conditional expression information for the distinguishing genes, because multiple biological processes contribute to the conditional expression given an arbitrary gene, but each distinguishing gene is effectively unique to its biological process. The computational task of discovering distinguishing genes is thus the task of locating the most extreme gene-gene conditional expression vectors in a space where the number of vectors and the number of dimensions are both equal to the number of genes (or probes), e.g., *g* = 20,000 or 50,000.

### Algorithm overview

The algorithm at the heart of our software tool is a modification and extension of an existing algorithm for the topic modeling problem [13]. We made changes to enhance execution speed and to reflect biological features that are not present in the generic topic modeling problem. Additionally, we implemented utility tools for direct access to the data sets within the NCBI Gene Expression Omnibus (GEO).

The algorithm for topic modeling [13] computes a *g* × *g* matrix ${\bar{Q}}_{i,j}=\mathrm{Pr}\left[f_{2}=j|f_{1}=i\right]$ of gene-gene conditional expression values from a *g* × *s* matrix *X* of linear (non-logarithmic) gene expression values, where *s* is the number of samples, via a matrix *Q* = *XX*^*T*^, where *X*^*T*^ is the matrix transpose of *X*. The matrix $\bar{Q}$ is then computed by rescaling each row vector of *Q* so that its entries sum to 1.0. A first pass then identifies tentative distinguishers. The first tentative distinguisher is the gene associated with that row vector of $\bar{Q}$ with the largest magnitude, computed using the standard Euclidean metric. In the general step, the $\bar{Q}$ matrix is then adjusted via a re-centering or matrix projection to be described momentarily, so as to map the row vector for the most recently selected tentative distinguisher to the origin. The next tentative distinguisher is then the gene whose row vector is most distant from the origin in this adjusted matrix, where all previous tentative distinguisher row vectors have been mapped to the origin.

The first tentative distinguisher's row vector is mapped to the origin by subtracting the row vector from each row vector of $\bar{Q}$. A subsequent tentative distinguisher's row vector $\vec{v}$ is mapped to the origin by orthogonal projection; a row vector $\vec{u}$ gets mapped to $\vec{u}-\frac{\vec{u}\cdot \vec{v}}{v\cdot \vec{v}}\vec{v}$. In this way, each tentative distinguisher is selected by virtue of being farthest from the hyperplane spanned by the previously selected tentative distinguishers. A second pass is performed so that each distinguisher is farthest not only from the previously selected tentative distinguishers, but also from the subsequently selected tentative distinguishers. This change in criterion sometimes causes a tentative distinguisher to be rejected in favor of a better distinguisher.

### Algorithm speed

One way to accomplish speed is to never actually compute or directly manipulate the *g* × *g* matrix of gene-gene conditional expression values, where *g* is typically 20,000 to 50,000 genes or probes. These operations would normally take time proportional to *g*^3^. Instead, the desired task is performed via matrix chain multiplication in a time that is proportional to the product *gsb*, where *s* is the number of samples and *b* is the number of biological processes sought. This strategy can reduce the computation time by several orders of magnitude. A second way of speeding up the calculations is by reusing sub-basis vector space projections in the algorithm's second pass for distinguishers. Instead of performing *b* distinct computations, which each project out *b* − 1 vectors of size *g* and which would require time proportional to *gsb* × *b*(*b* – 1) in total, we reuse sub-computations in a way that accomplishes the task in time proportional to *gsb* × *b* log_2_(*b* – 1). The gain from this part is usually around one order of magnitude.

### Algorithmic features specific to gene expression data

The biologically motivated modifications are made to reflect that low gene expression values are relatively noisy and strongly expressed genes are considered to be more useful as distinguishers than weakly expressed ones, and that there is value in having multiple genes (runners-up) to distinguish each biological process. To model that genes with low expression are poor distinguishing genes, we mathematically spike all biological samples with a low-level expression for each gene. For genes with generally high expression this has negligible effect on the subsequent analyses, but for genes with generally low expression it adequately hides the possibility that the gene is expressed in only one biological process. Genes with low to moderate expression are hindered but not eliminated from consideration. We set the amount of expression spike to be the 75 percentile among all positive expression values by default, but this can be adjusted to any value.

After a greedy-approach first pass, the algorithm has a tentative list of distinguishing genes to identify distinct biological processes. The quality of each distinguishing gene on this list is then gauged by the distance of its conditional expression vector from the hyperplane spanned by the conditional expression vectors of other distinguishing genes, with greater distances indicating stronger distinctiveness for the biological process. However, there may be other genes that are distant from the hyperplane in the direction of the tentatively identified distinguishing gene. Ranked by the distance from the hyperplane, the user-requested number of most-distant genes are returned. Note that a tentatively identified distinguishing gene will not necessarily be the most distant from its corresponding hyperplane, even though it is the direction of the tentatively identified distinguishing gene from the hyperplane that is used for gauging distances.

### Running the algorithms

CellDistinguisher runs in the software environment provided by The R Project for Statistical Computing. The software is configured to use linear (i.e., not log2 transformed), non-negative gene expression values of the mixed samples in an R matrix or as read from a tab-separated text file, with samples in columns and genes in rows. In the case of a file, the first line is expected to contain the sample names in quotes, and the first column of each row should hold the name of a probe or gene. The software also allows the retrieving of NCBI GEO data sets directly.

To compare our deconvolution approach to existing and published methods, we used the R package CellMix [10, 11]. The partial deconvolution methods lsfit and qprog implemented in CellMix provide estimates of cell-type proportions in a mixed sample given the expression profiles of the pure cell types. The opposite is performed by cs-lsfit: it derives the expression signatures given the cell type proportions. The semi-supervised algorithms ssKL and ssFrobenius use distinguisher ("marker") genes as input to determine sample composition. The performance of CellDistinguisher coupled with ssKL or with our in-house partial deconvolution tool was tested and compared to lsfit, qprog, cs-lsfit and deconf.

### Data sets analyzed

We analyzed two data sets for which complex samples were simulated by mixing cell-type or tissue-specific gene expression values in controlled proportions to demonstrate the efficiency and accuracy of the algorithm. We also applied CellDistinguisher to three real gene expression data sets described below.

#### Simulated mixed samples from normal human tissues

**Fig 1:**
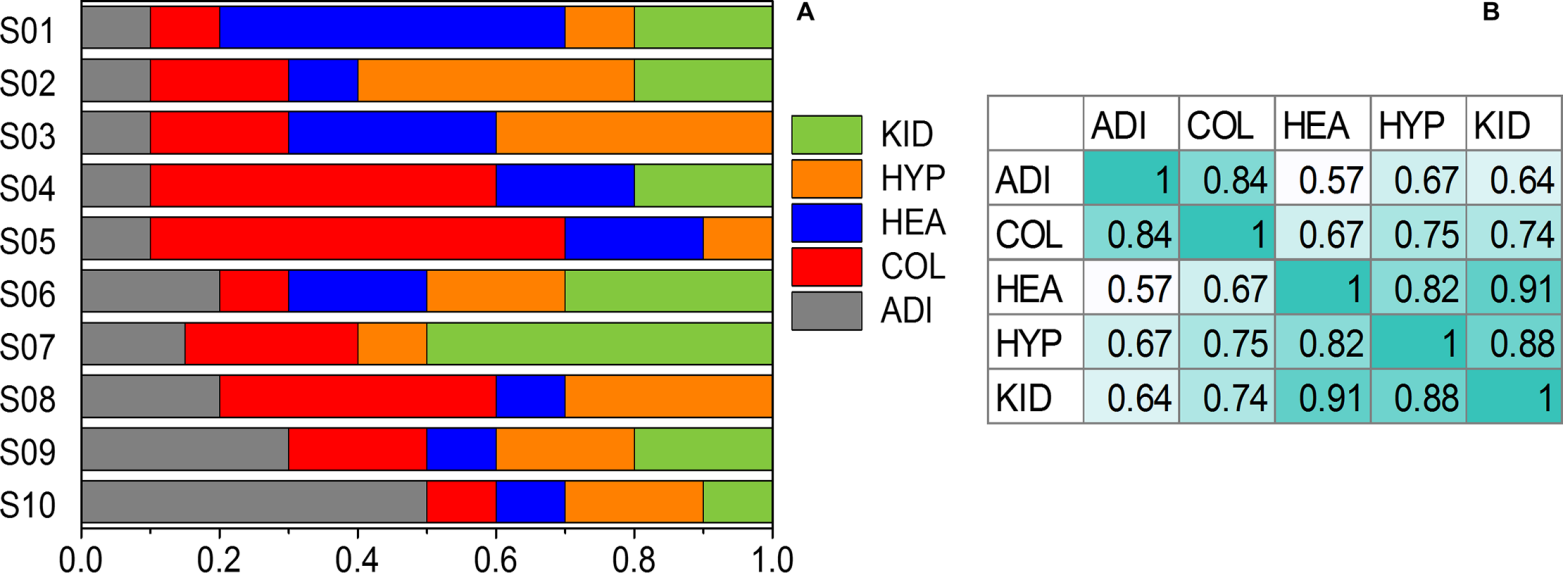
Composition of synthetic samples generated from RNA-Seq Atlas tissue data. (A) Visual representation of mixed sample compositions. (B) The correlations between the noiseless gene expression signatures of the tissues included in testing. Notations: ADI–adipose, COL–colon, HEA–heart, HYP–hypothalamus, KID–kidney.

Gene expression data from pooled normal tissues was obtained from the RNA-Seq Atlas [14]. Ten mixed samples were generated by combining the expression values of known genes from five tissues (adipose, colon, heart, hypothalamus and kidney) using various combinations of cell type fractions (Fig 1A, S1 Table). Out of the total number of 21,399 genes, 2,240 had zero expression values in each of the five selected tissue types and were excluded from our analysis. We considered this to be a moderately easy test case because the cell types are relatively distinct from each other with correlations between signatures in the 0.57–0.91 range (Fig 1B). In successive simulated experiments, an increasing amount of noise was added to the expression values of the noiseless mixed samples. For a noiseless expression value x, noise was introduced by drawing a random number from a log-normal distribution with mean x and standard deviation (SD) of σx, where the relative standard deviation σ ranged from 0% to 100% through successive experiments. Three replicates were generated for each simulated mixed data set.

#### BrainSpan data set with simulated mixed samples

The BrainSpan Atlas of the Developing Human Brain contains RNA-seq and microarray expression data from 26 brain sub-regions of 35 healthy individuals, each individual having 8–10 regions profiled [15, 16]. To better control the data quality, we used the expression data of only those samples and genes that were present in both the RNA-Seq and microarray data sets, resulting in 433 samples and 16,718 genes. Transcriptional differences across brain samples in this database reflect both differences in developmental stages and differences in compositions due to varied proportions of brain cell types among distinct brain regions [17]. In our analysis, we aimed at using brain regions with distinct expression patterns reflecting differences in underlying cell types. To limit the effect of developmental differences on the gene expression values, we restricted the analysis to adult samples of at least 18 years old. We also performed heatmap-type visualization and K-Means clustering to compare the gene expression patterns of different brain regions and select four relatively distinct regions for further analysis: primary visual cortex (V1C), cerebellar cortex (CBC), mediodorsal nucleus of thalamus (MD) and striatum (STR).The details of generating mixed samples based on a Dirichlet distribution of brain region proportions as well as adding realistic variance to the expression values are described in the Supplementary Methods in S1 File.

#### Mixtures of rat liver, brain, and lung tissues

The GSE19830 data set includes gene expression measures from mixtures of rat liver, brain, and lung cells in different proportions *[18]*. There are 3 pure samples and 11 mixtures in the set. Each experiment was done in triplicate, yielding 42 gene expression profiles in total of which we used the 33 for the mixtures. The data set was downloaded from the NIH NCBI Gene Expression Omnibus (GEO) [19, 20].

#### Early stages of human B cell development

The GSE14714 data set includes gene expression measures from 5 different stages of B cell development in human. There are 6 replicates for each stage, yielding 30 gene expression profiles in total [21].

#### Yeast cell cycle

We downloaded the microarray gene expression data set of Cho et al. from the Saccharomyces Genome Database [22, 23]. These data were generated to characterize the genome-wide changes in the mRNA levels while the budding yeast S. cerevisiae undergoes the mitotic cell cycle. Samples were obtained at 17 time points from 0 to 160 minutes, roughly corresponding to one and a half to two rounds of the yeast cell cycle. CellDistinguisher was applied to this dataset with the aim of retrieving the genes whose expression levels increased and decreased at various phases of the cell cycle, capturing the predominant cellular processes of the various phases.

## Results

### Simulated mixed samples from normal human tissues

**Fig 2:**
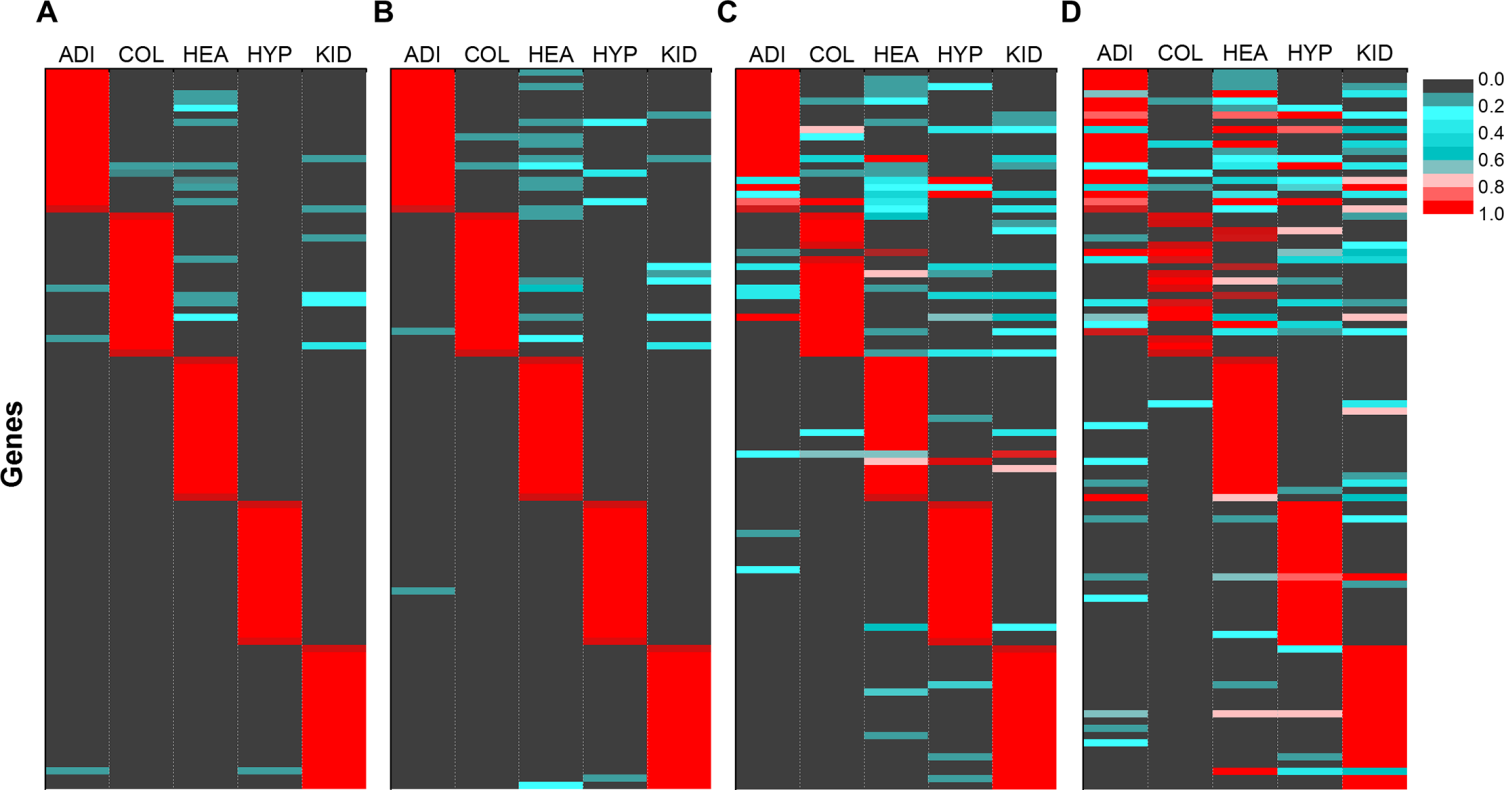
Distinguisher genes were highly expressed in specific tissue types. Gene expression values of the top 20 distinguisher genes for each tissue/cell type are shown in the heat maps at noise levels of 0%, 20%, 40%, and 60% relative standard deviation in panels A to D, respectively. Expression values were normalized from 0 to 1 (colored grey to red) for each gene across the five tissue/cell types (ADI = adipose, COL = colon, HEA = heart, HYP = hypothalamus, KID = kidney).

CellDistinguisher correctly picked genes that were differentially expressed at high levels in individual cell types at low noise (0–20% SD, Fig 2A and 2B). Not surprisingly, as the error level increased to 40–60% SD, the number of incorrectly identified cell type markers increased (Fig 2C and 2D). At 100% SD, distinguisher identification became unreliable, similar to randomly choosing genes.

**Fig 3:**
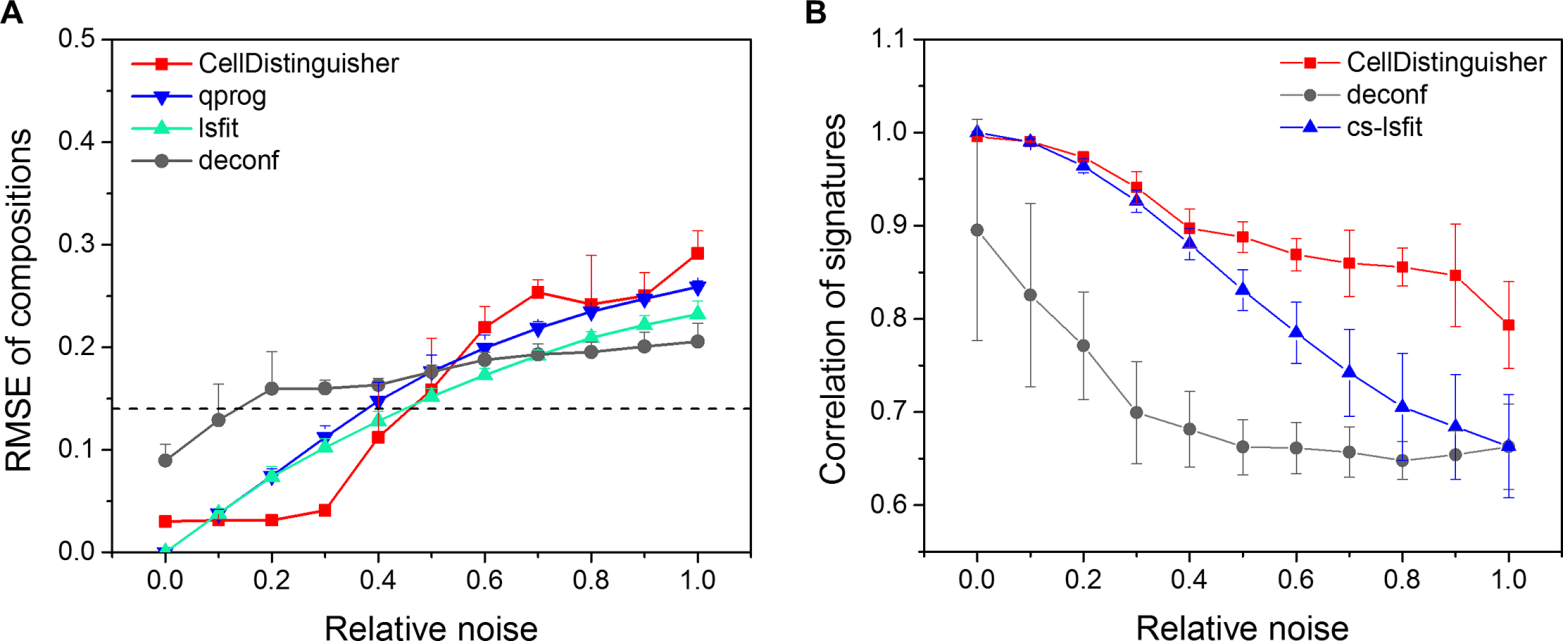
Performance of CellDistinguisher on mixed human tissue dataset compared to other methods. (A) Predicting sample composition and (B) cell type signatures in mixed human samples as a function of the amount of noise added to the expression data. CellDistinguisher was compared to the partial deconvolution methods qprog, lsfit and cs-lsfit and the complete deconvolution method deconf.

The top 10 distinguisher genes for each cell type were passed on to the deconvolution algorithm ssKL to predict the composition of the mixed samples. Sample composition was also calculated with the partial deconvolution algorithms lsfit and qprog, as well as the complete deconvolution tool deconf. The average root mean squared error (RMSE) between the expected and predicted cell type fractions across the 10 mixed samples was calculated to quantify the accuracy of the sample composition prediction. For assessing the accuracy of the pure cell type signature predictions, the Pearson correlation coefficient between the known and predicted signatures was also calculated. At low noise levels, CellDistinguisher combined with ssKL performed just as well or even better than the other deconvolutions algorithms in calculating the correct sample compositions (Fig 3A). As the noise level reached a 40% standard deviation, the introduction of an increasing number of incorrect distinguishers among the top 10 per cell type led to a gradual deterioration of the performance reflected by the increasing RMSE in cell composition prediction. Regardless of the noise level, CellDistinguisher was superior to deconf in identifying the best possible cell type signatures even though the cell type fractions predicted by deconf were somewhat closer to the expected values with high noise levels (Fig 3B). The likely explanation for this observation is that although the numbers representing the fractions were close to the expected values, the corresponding cell types assigned to these fractions by deconf were inconsistent. Attempts to improve the performance of deconf by forcing it to run through a larger number of iterations or using either of its two implementations in CellMix were unsuccessful. The algorithm converged very quickly, performing very few steps of iterations (observed number was always < 20) even if the maximum number of iterations was set much higher. CellDistinguisher performed equally well at low noise levels as the partial deconvolution method cs-lsfit at predicting cell type signatures. At higher noise levels, CellDistinguisher was more accurate than cs-lsfit.

**Fig 4:**
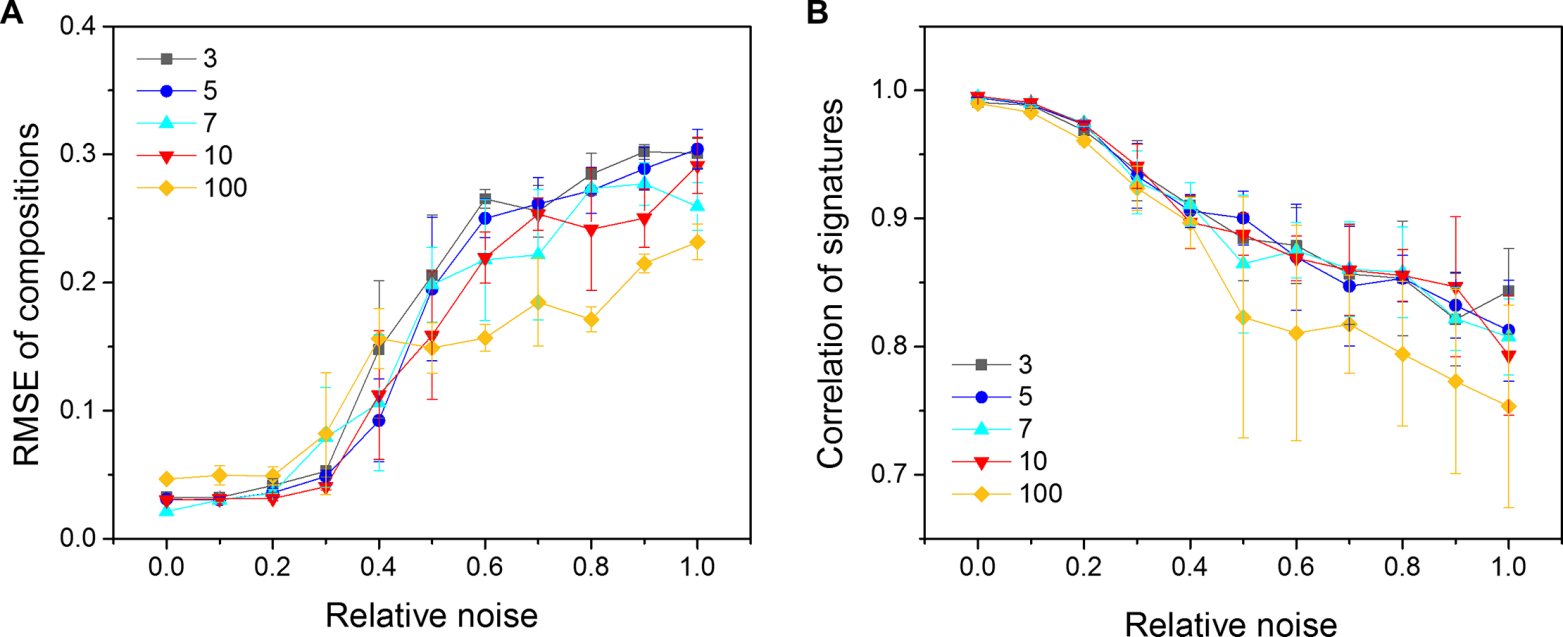
Distinguishing gene set size effect on predicting sample composition. (A) The performance of CellDistinguisher combined with ssKL in predicting sample composition and (B) cell type signatures as a function of noise and the numbers of distinguisher genes passed on to ssKL using the artificially mixed human tissue samples.

We tested the influence of the number of distinguisher genes passed from CellDistinguisher on to ssKL on the accuracy of the cell composition prediction. At low noise level, a maximum number of 10 distinguishers resulted in better performance than using up to 100 distinguishers (Fig 4A). For very noisy data, a larger number of distinguishers seemed to offer some advantages in terms of predicting the cell composition. However, the decline in correlations between predicted and expected cell type signatures indicated that the likelihood of including false distinguishers increased when larger sets of genes were used (Fig 4B).

### BrainSpan data set

Four regions were selected for the deconvolution analysis as the representatives of four different tissue/cell types in the brain regions from BrainSpan: V1C - primary visual cortex, CBC—cerebellar cortex, MD—mediodorsal nucleus of thalamus and STR–striatum.

**Fig 5:**
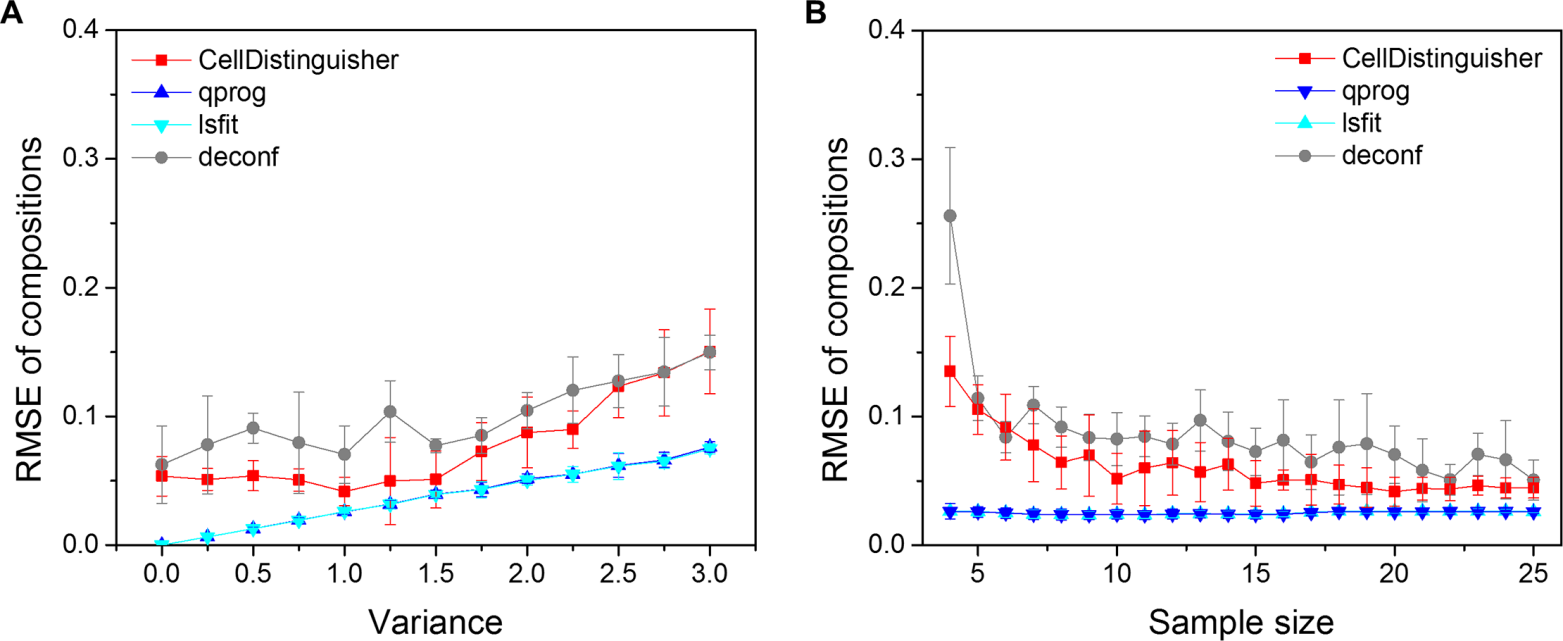
Performance of CellDistinguisher on the BrainSpan dataset compared to other methods. (A) Comparison of sample composition predictions by CellDistinguisher, qprog, lsfit and deconf on the BrainSpan dataset as a function of noise expressed as the magnitude of variance at sample size 20 and (B) the number of samples at variance 1.

**Fig 6:**
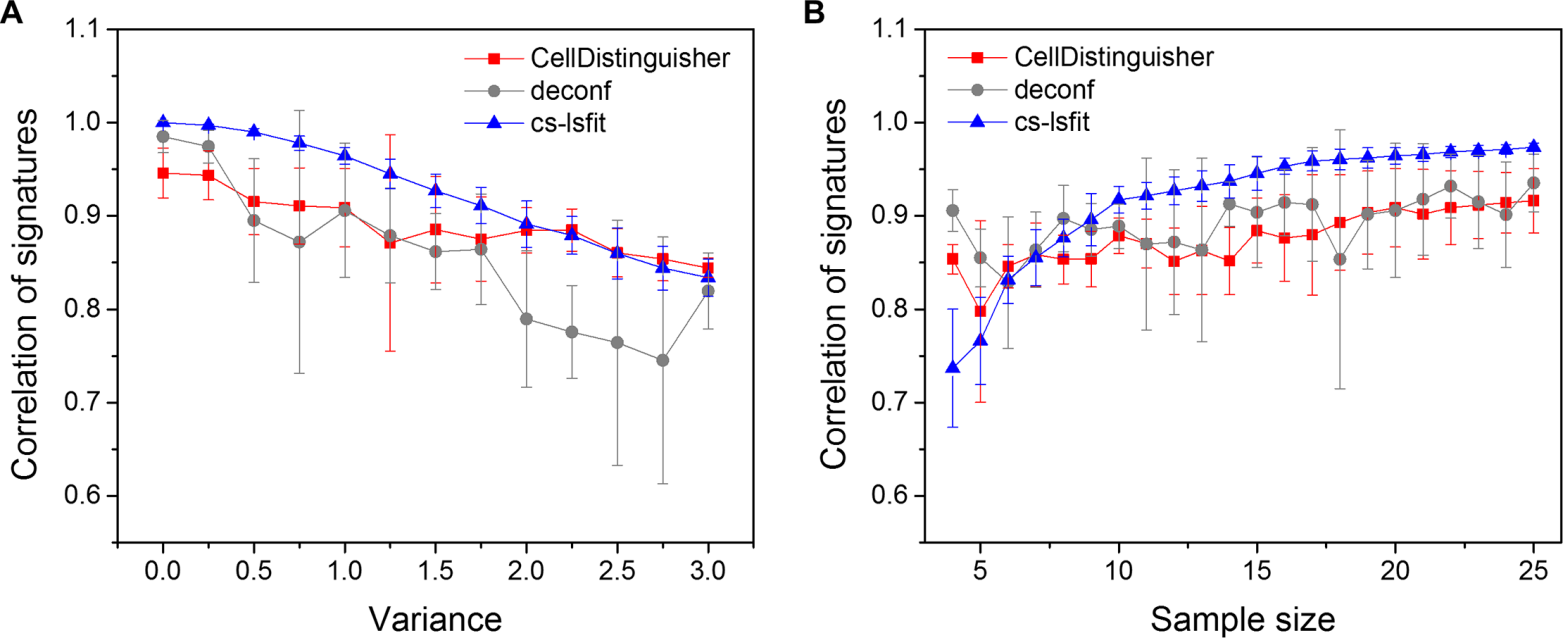
Comparison of signature predictions by CellDistinguisher, deconf and cs-lsfit. (A) Correlation of predicted and expected signatures versus magnitude of variance at sample size 20 and (B) the number of input mixed samples at variance 1.

By using the simulated mixed samples with realistic means and variances estimated from the RNA-seq data of the selected four regions, we examined and compared the performance of CellDistinguisher to the other deconvolution methods. The partial deconvolution methods lsfit, qprog and cs-lsfit showed near perfect estimation of proportions and signatures when the variance magnitude was equal to 0 (no variance), regardless of the number of samples (Figs 5 and 6). As the magnitude of the variance increased, the estimation became more difficult, even for the partial methods, as indicated by the increased root mean squared errors (RMSE) of proportions and decreased correlations of true and estimated signature levels. Since no cross-sample information was needed for partial deconvolution of proportions, the number of samples had practically no effect on the performance of lsfit and qprog (Fig 5B).

Complete deconvolution is a much tougher problem compared to partial deconvolution, as no extra information is provided to infer the sample signatures or proportions. The existing method, deconf, predicted the sample compositions with much larger errors, showing higher RMSE of proportions and lower correlation of signatures compared to the partial methods (Figs 5 and 6). The CellDistinguisher results were more accurate in composition estimation compared to deconf (Fig 5). The accuracy of CellDistinguisher in signature estimation was similar to that of deconf at low noise, but surpassed it at high noise levels (Fig 6A). Increasing the number of input samples improved the prediction accuracy, as expected, for both proportion and signature predictions. CellDistinguisher was consistently better than deconf in composition prediction regardless of the sample size (Fig 5B), while their performance in signature prediction was similar on this dataset (Fig 6B).

**Fig 7:**
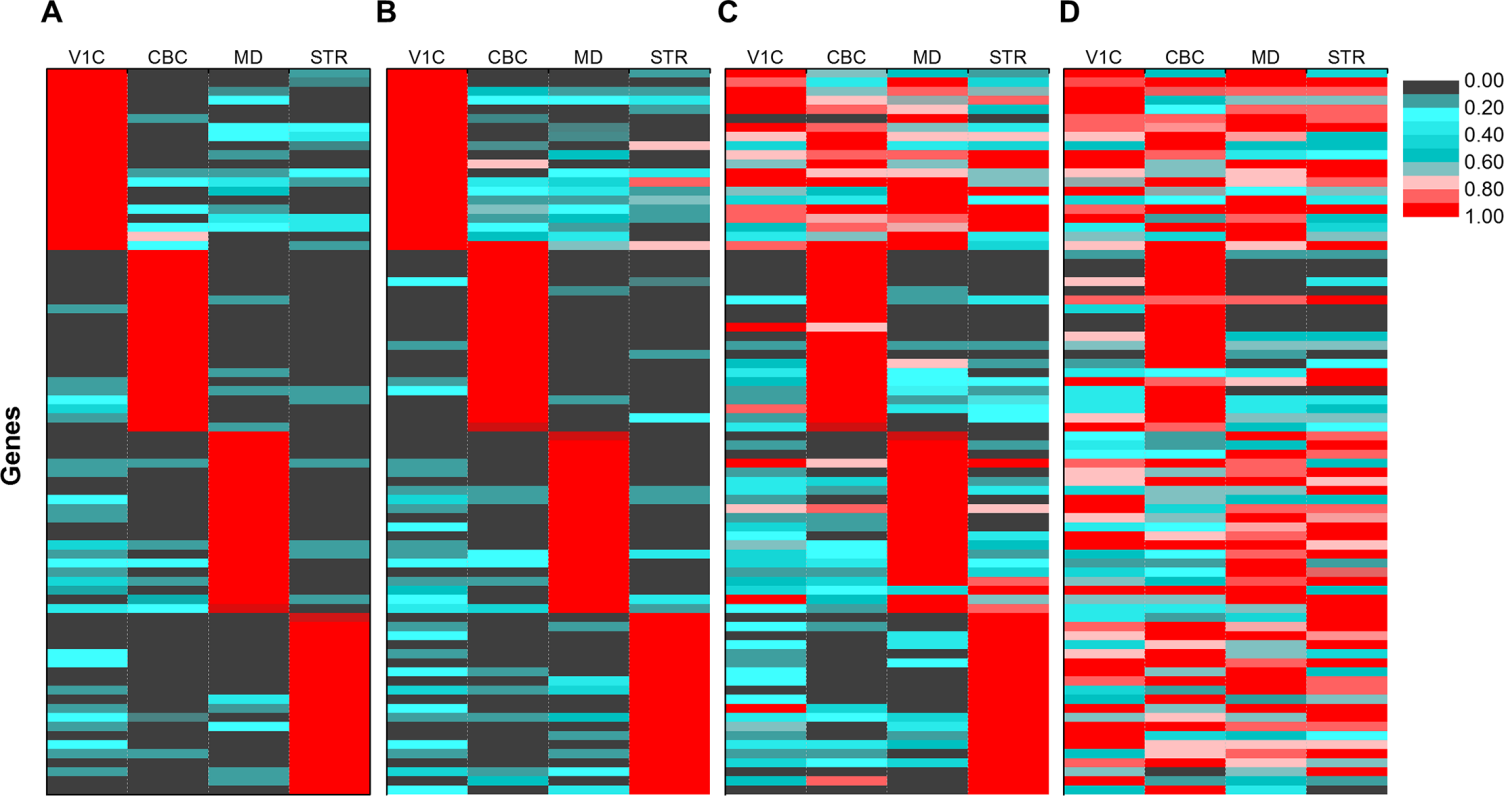
CellDistinguisher identified genes differentially expressed in specific brain cell types. The expression values of the best 20 distinguishers found by CellDistinguisher for the four brain cell types at magnitudes of variance of 0, 1, 2 and 3 in panels A through D, respectively. Columns represent the four different known cell/tissue types used to generate the synthetic mixed samples. The color-coded groups of lines represent the gene sets identified as the distinguisher genes of the 4 cell types. Values were normalized from 0 to 1 for each gene across the four cell types. The number of mixed samples used as input was 20.

To examine the accuracy of the identified distinguisher genes, we plotted the gene expression values of the 20 best distinguishers found in the four cell types at the variance levels 0, 1, 2 and 3 (Fig 7). Generally, CellDistinguisher picked distinguisher genes which were predominantly expressed in a single cell type. At high variance levels (e.g. 2), most distinguishing genes were found to be mainly expressed in one cell type, but a few of them also had non-negligible expression levels in two or more other cell types (Fig 7C). As the variance increased further, the recognizable cell-specific patterns gradually disappeared (Fig 7D).

### Mixtures of rat liver, brain, and lung tissues

We computationally analyzed the gene expression profiles of the 11 mixed samples of this data set available in triplicates. Up to 100 distinguishers were identified for each of the three cell types. They are reported in descending order of quality as a distinguisher in the supplementary material (Supplementary Table 2 in S1 Table). These were then used to compute the tissue composition in each sample using our in-house deconvolution algorithm. Based upon the resulting accurate deconvolution (to be described next), it was concluded that these genes represented biological processes specific to liver, brain, and lung, respectively. These genes might play roles in biological processes that can occur in tissues other than these three, but they are highly selective in distinguishing liver, brain and lung. As additional support for their correct assignment as cell-specific marker genes, the distinguishers for each group were annotated and analyzed using the web-based functional annotation tool David [24, 25]. The three groups were significantly enriched in liver, brain and lung specific genes, respectively. From the first group of distinguishers, 99 were recognized as valid David gene symbols, and 77 were annotated as specifically expressed in the liver (p-value 1.5E-53) based on UniProt-derived information [26]. From the second group of distinguishers, 85 were recognized as valid gene symbols, out of which 61 were annotated as specifically expressed in the brain (p-value 5.6E-30). Finally, from the 71 distinguishers of the third category that were recognized as valid David gene symbols, 28 were labeled as lung-specific (p-value 2.2E-16). The gene lists and their annotations are included in the supporting information (Supplementary Tables 3–5 in S1 Table).

**Fig 8:**
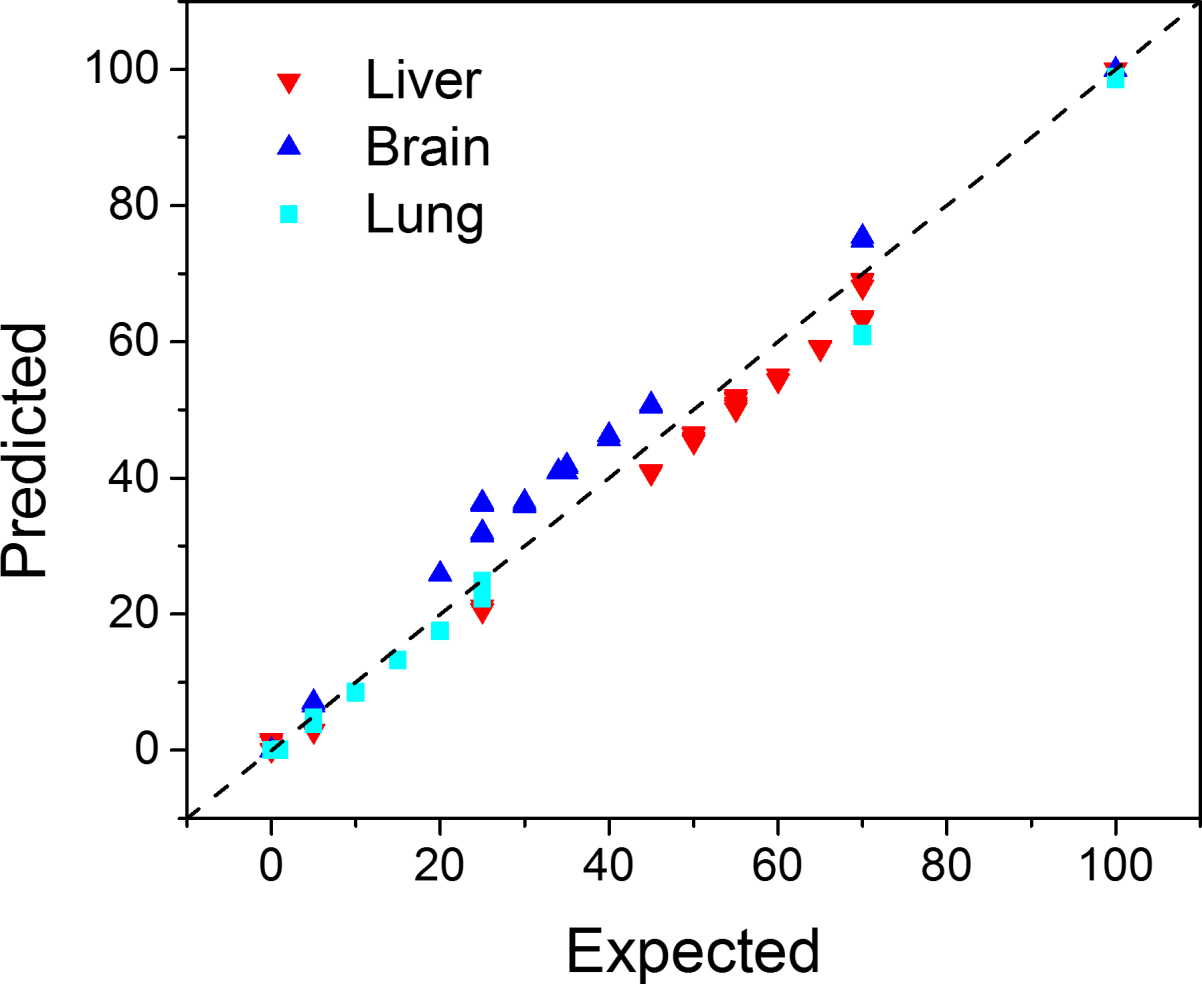
CellDistinguisher accurately predicted cell type composition in the rat tissues. The Pearson correlation coefficient between expected and predicted compositions was 0.99. Additionally, we compared the gene expression signatures, which were assigned to the biological processes by the deconvolution, with the gene expression signatures of the pure samples, each averaged over its triplicates. The cosine (*x* ⋅ *y*) / (|*x*| |*y*|) of the concatenation of the three computed signatures with the concatenation of the three pure-sample signatures is 96.8% indicating that the inferred gene expression signatures are highly representative of the pure-sample gene expression signatures.

We found excellent correlation between the compositions calculated with CellDistinguisher and expected cell type ratios, with an overall correlation of 0.99 (Fig 8). The deconvolution results in the form of expected and predicted cell type ratios for each sample are presented in Supplementary Table 6 in S1 Table. The variation in sample composition from replicate to replicate was negligible. However, the concordance between the original author-provided labels that indicate a sample's proportions did not always perfectly match the derived proportions. Given the high reproducibility, we speculate that the discrepancy might be due to inconsistencies between what the author-supplied labels mean (relative amount of tissue homogenate) and what the deconvolution produced as sample composition (relative amount of RNA). This is especially plausible given that the deconvolution results consistently underestimated the proportions of the liver and overestimated the proportions of the brain. Our composition prediction agrees very well with the one reported by Wang and colleagues using the CAM algorithm both in the overall extent of the correlations with the expected proportions as well as the slight but systematic overestimation in brain and underestimation in liver [12]. The correlation coefficient between the composition predictions of the two methods was found to be 0.995. A detailed description of the comparison between CellDistinguisher and CAM can be found in the Supplementary Material in S1 File.

### Early stages of human B cell development

**Fig 9:**
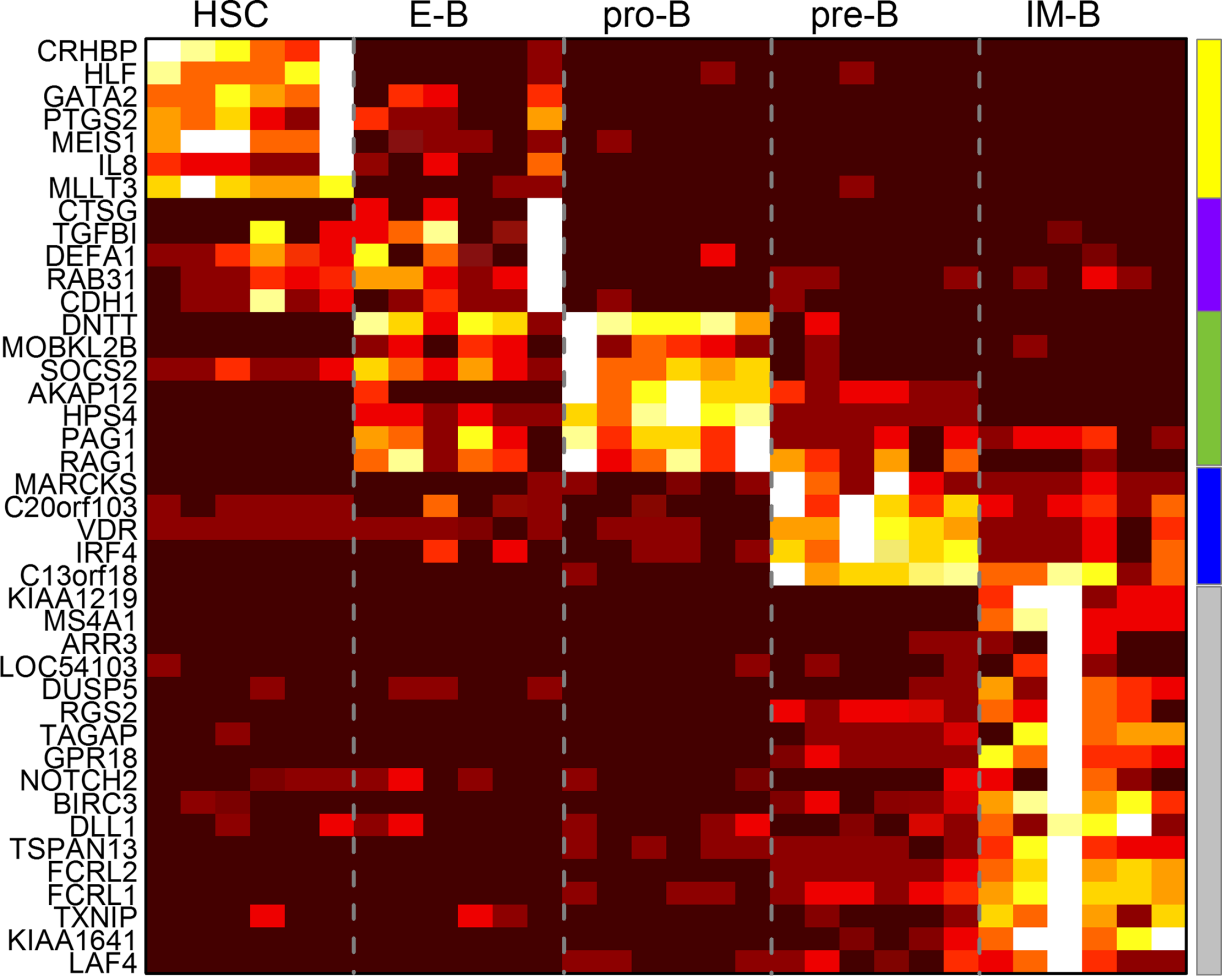
Expression profiles of distinguisher genes in the five developmental stages of B cells. The expression values were normalized from 0 to 1 (from dark to light) for every gene across the samples. The colored bars on the right indicate the groups of genes designated as distinguishers of the different stages.

From this data set, we extracted six replicates of gene expression measures from 5 different stages of B cell development in human, resulting in 30 gene expression profiles in total. The algorithm was set up to discover up to 20 distinguishers for 5 categories. Hystad et al. [21] provide cell surface markers detectable by flow cytometry to characterize the stages of B cell development. The CellDistinguisher results, which are computed in minutes without relying on the curation of years of accumulated research, indicate that B cells can alternatively be classified by the expression values of several genes that are differentially expressed at the various stages of the development (Fig 9, Supplementary Tables 7, 8 in S1 Table). Based on David annotations, many of these distinguishers are known to be related to stage-specific immune processes, B cell specific, hematopoietic, inflammatory and cell developmental functions (Supplementary Table 9 in S1 Table).

### Genes with alternating expressions during the yeast cell cycle

**Fig 10:**
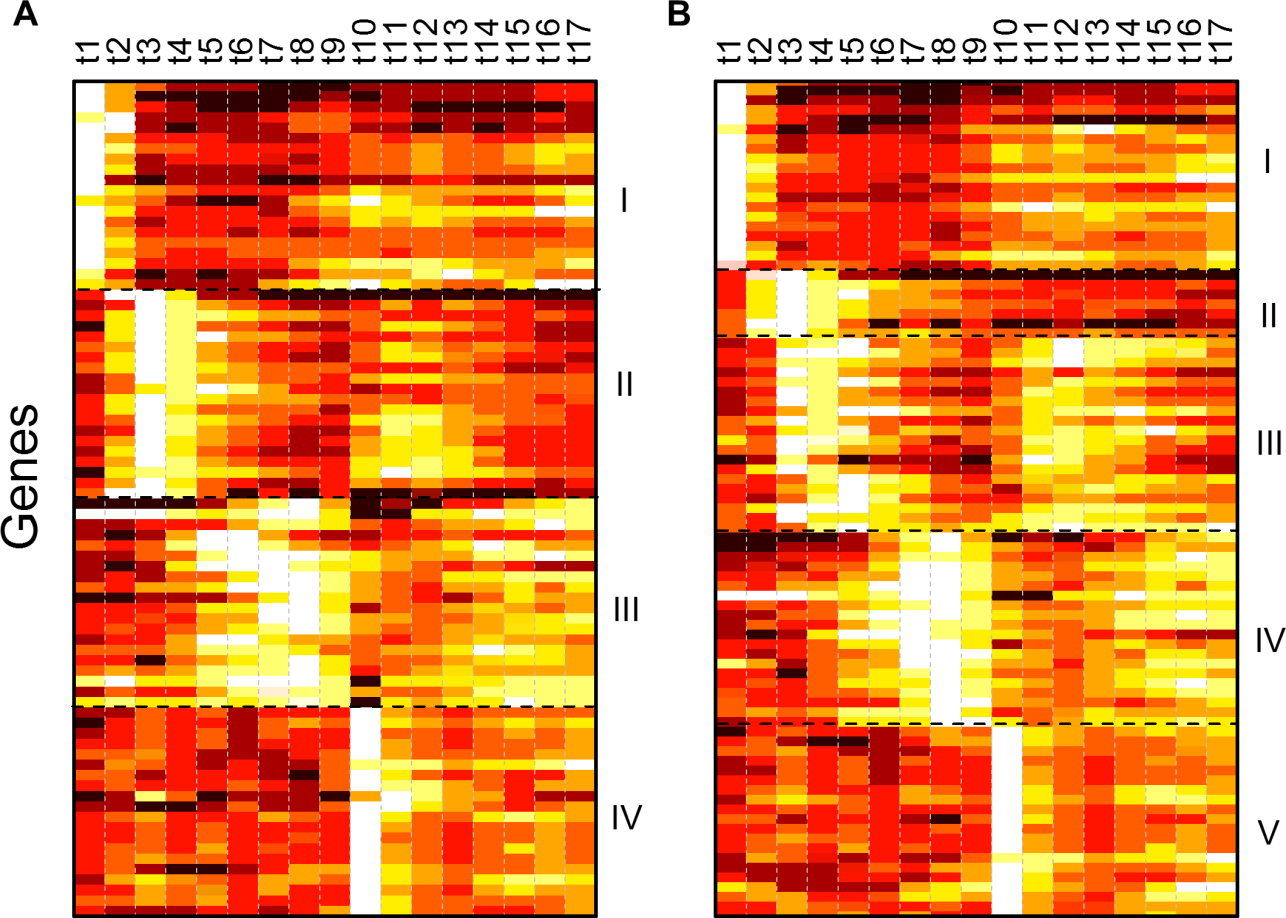
Expression profiles of distinguisher genes along the yeast cell cycle. Yeast genes reaching maximum expression levels at different time points during the mitotic cell cycle inferred from the Cho data set with CellDistinguisher grouped in 4 and 5 gene categories in panels A and B, respectively. Up to 20 top ranking genes were included per category. Expression values were normalized from 0 to 1 (dark to light) across samples for each gene individually.

CellDistinguisher was applied to the yeast cell cycle data set setting it up to retrieve up to 100 genes grouped into 2 to 8 categories. Since the samples differed from each other primarily in the phase of the cell cycle they were trapped into, the algorithm identified groups of genes with expression values peaking at different time points along the cycle (Fig 10). A clean pattern of separation could be observed at 4 groups (Fig 10A, Supplementary Table 10 in S1 Table). Asking for an additional category resulted in refining and splitting group II further, while the others remained mostly the same (Fig 10B). Not surprisingly, defining more categories lead to further refinement of the gene clusters by cycling patterns, with some clusters having only a handful of distinguishers.

## Discussion

When either cell type signatures or sample compositions are available, partial deconvolution methods can be used to quantitate the other. These methods are very easy to use, fast and accurate. However, for more challenging questions, when no pure component signatures or sample compositions are available, complete deconvolution is employed to solve the problem. CellDistinguisher facilitates complete deconvolution by identifying genes whose expression levels point to distinguishable biological processes or cell types. Paired with a deconvolution algorithm that takes marker gene lists as input, ssKL in our testing, complete deconvolution is achieved with the precision and accuracy of partial deconvolution methods. CellDistinguisher's performance with our in-house deconvolution tool (DeconvolutionByDistinguishers) was similar.

Simulated mixtures of normal tissues showed that error levels in gene expression values below a 40% relative standard deviation are acceptable. Above this noise level, the accuracy of predicting cell type composition is poor regardless of which approach is used. Interestingly, even at these high error rates, the cell type signatures were relatively faithful to the expected signatures, especially for the more distinct cell types. For the multi-tissue simulated data set built from RNA-seq Atlas data, the worst RMSE for a uniform distribution mixture would be 0.14. Predictions with RMSE values above this level can be considered worse than random. CellDistinguisher coupled with ssKL crossed this threshold slightly above an error level of 40% relative standard deviation. Similar performance was observed with the partial deconvolution algorithms lsfit and qprog. The complete deconvolution method deconf broke down much faster, at approximately a 10% relative standard deviation.

The maximum number of distinguishers to be used per biological process depends on the size and quality of the data set analyzed. Intuitively, one would be tempted to use as many distinguishers as possible to get the best performance. However, in our experience that is not necessarily the best strategy. For our test sets, a maximum number of 10 to 20 distinguishers proved to be a good choice.

In many cases, the list of distinguishers itself is the desired information. However, the distinguishers can be leveraged to perform gene expression deconvolution of experimental data. Deconvolution provides both the composition of each sample in terms of the cell types or biological processes present and the gene expression signatures that characterize these components. We have provided a fast functionality for this purpose which can be invoked with DeconvolutionByDistinguishers. Multiple other approaches for deconvolution based on gene lists are publicly available and can be combined with CellDistinguisher to compute sample composition. In addition to our in-house method, we tested and can recommend ssKL and ssFrobenius provided by the CellMix package [10, 11].

Much as with choosing the number of clusters in a clustering problem, it can be difficult to know how many different biological processes can be inferred from the data. The number should be at least 2 and should not be more than the number of samples. There is an internal parameter computed by CellDistinguisher whose value might provide some guidance in this regard. It is called “bestLengths” and it represents the distance of a particular distinguisher from a hyperplane defined by the top distinguishers of the other biological processes. It is somewhat analogous to the standard deviation that is associated with each component in a principal component analysis. Our experience is that a sudden drop of the values in the first row of the bestLengths (hyperspace distances associated with best distinguishers) indicates the point at which the value of increasing the number of biological processes is questionable.

CellDistinguisher is not restricted to deriving the composition of mixed samples, as illustrated by its application to the human B-cell development and the yeast cell cycle data sets. It performs something analogous to a principle component analysis by identifying the genes whose expression best distinguish the relatively homogeneous samples.

By default, CellDistinguisher tends to pick distinguishers from highly expressed genes. Parameter values can be adjusted to allow genes with overall lower expression values to be considered as well. One feature that we plan to implement in the feature is the negative discrimination that will allow a relative lack of expression to be selected as a subpopulation distinguisher.

## Software availability

The R implementation of CellDistinguisher is available in the GitHub repository (https://github.com/GeneralElectric/CcellDdistinguisher)

## Supporting information

S1 Table Sample compositions and distinguisher genes for the analysed datasets. (1) The fractions of various cell types in the mixed rat samples. (2) Rat sample compositions. (3) Rat tissues–best distinguishers. (4) Rat liver distinguishers. (5) Rat brain distinguishers. (6) Rat lung distinguishers. (7) B-cell compositions. (8) B-cell profiles–best distinguishers. (9) B-cell best distinguisher annotations. (10) Yeast cell cycle: Normalized linear expression values of top 20 distinguishers for 4 classes/phases.
(XLSX)

S1 File Supplementary methods and results. Algorithmic differences between CellDistinguisher and CAM. Description of the BrainSpan data set with simulated mixed samples. Comparison of the performance of CellDistinguisher and CAM on three independent datasets.
(PDF)
