# Supplementary material for: Computational de novo discovery of distinguishing genes for biological processes and cell types in complex tissues
Source: PLoS One. 2018 Mar 1;13(3):e0193067. doi: 10.1371/journal.pone.0193067 (PMC5832224; doi:10.1371/journal.pone.0193067)
Supplement: S1 File — Algorithmic differences between CellDistinguisher and CAM. Description of the BrainSpan data set with simulated mixed samples. Comparison of the performance of CellDistinguisher and CAM on three independent datasets. (PDF) [file pone.0193067.s002.pdf]

Computational de novo discovery of distinguishing genes for biological processes and cell types in complex tissues

Lee A. Newberg, Xiaowei Chen, Chinnappa D. Kodira, and Maria I. Zavodszky

Content of supplementary material

S1_Table.xlsx

Supplementary Tables

1. The fractions of various cell types in the mixed samples
2. Rat tissue compositions
3. Rat tissues – best distinguishers
4. Rat liver distinguishers
5. Rat brain distinguishers
6. Rat lung distinguishers
7. B-cell compositions
8. B-cell profiles – best distinguishers
9. B-cell best distinguisher annotations
10. Yeast cell cycle: Normalized linear expression values of top 20 distinguishers for 4 classes/phases

S1_File.pdf

Supplementary Methods

Algorithmic differences between CellDistinguisher and CAM
BrainSpan data set with simulated mixed samples

Supplementary Results

Comparison of the performance of CellDistinguisher and CAM on three independent datasets

Supplementary methods

Algorithmic differences between CellDistinguisher and CAM

Similar to the earlier approach of topic modeling [1], which is the inspiration for CellDistinguisher, Wang at al. provides CAM, which looks for cell type markers by looking for genes whose expression vectors (across samples) form the vertices of a simplex approximately enclosing the expression vectors of all genes [2]. The approach they use to find these vertices differs from that of Arora et al. and CellDistinguisher. CAM first eliminates genes whose signal intensity (vector norm) is lower than 5% (noise) or higher than 95% (outlier) of the mean value over all genes. It then performs a dimension reduction to effectively reduce the number of samples via a principal components analysis of the gene expression information. Another dimension reduction follows to effectively reduce the number of genes via affinity propagation clustering of the gene expression vectors, so that the number of gene clusters is m. The gene centers of these clusters are selected b at a time and evaluated as being suitable for vertices of a simplex that approximately includes the remaining $m - b$ gene cluster centers. The best set of b clusters is used as the solution for b biological processes, and the genes in the clusters are deemed to be the distinguishers. A minimum description length criterion determines the optimal value of b.

In contrast to CAM, CellDistinguisher allows the consideration of more genes by employing a more lenient filter for eliminating outlier expression, deeming a gene to be an outlier if its expression in one sample dominates all the other samples. A tunable parameter enables to user to modify what constitutes an outlier based on familiarity with the biological data type to be analyzed. CellDistinguisher employs an expression spike to deemphasize the distinguishing quality of genes with low expression, without explicitly eliminating them. This feature is also tunable because the dynamic range of different data types can be different. CellDistinguisher does not employ the approximation of dimension reduction either in the sample space (via principal components analysis) or in the gene space (via the results of a clustering approach). Distinguishers are identified without the need for exhaustive enumeration of sets of size b. CellDistinguisher does not attempt to determine the appropriate number of biological processes b, though some of its output is useful in a manual determination.

BrainSpan data set with simulated mixed samples.

The BrainSpan Atlas of the Developing Human Brain contains RNA-seq and microarray expression data from 26 brain sub-regions of 35 healthy individuals, each individual having 8-10 regions profiled [3, 4]. To better control the data quality, we used the expression data of only the samples and genes that were present in both RNA-Seq and microarray data sets, keeping 433 samples and 16718 genes. Transcriptional differences across brain samples in this database reflect both differences in developmental stages, as well as differences in compositions due to varied proportions of brain cell types among distinct brain regions [5]. In our analysis, we aimed at using brain regions with distinct expression patterns reflecting differences in underlying cell types. To limit the effect of developmental differences on the gene expression values, we restricted the analysis to adult samples of at least 18 years old. We also performed heatmap-type visualization and K-Means clustering to compare the gene expression patterns of different brain regions and select relatively distinct four regions for further analysis.

Noiseless mixed samples were simulated with different proportions of selected brain regions:

$$X_{jk} = \sum_{i=1}^{n} \mu_{ij}\, p_{ik} \quad (1)$$

where X_{jk} is the expression value of gene j in mixed sample k, μ_{ij} is the mean expression of gene j in brain region i, and p_{ik} is the proportion of region i in sample k. Each cell type signature value μ_{ij} for a region i was defined as the mean expression value of the gene j across the samples from that region.

To ensure a wide variety of mixed samples, we simulated the brain region proportions based on a Dirichlet distribution with a hyper-parameter equal to 3 for each dimension:

$$(p_{1k}, \ldots, p_{nk}) \sim \mathrm{dirichlet}(a, \ldots, a), a = 3 \quad (2)$$

where n is the number of cell types in a sample and $(p_{1k}, \dots, p_{nk})$ are the real proportions for cell types in sample k. With the choice of $a = 3$, the realized proportions $(p_{1k}, \dots, p_{nk})$ are likely to be nearer to uniform than would arise with a choice of $a = 1$ and thus represent a more challenging test case than $a = 1$ would.

To obtain a realistic variance for the expression values, we calculated the mean and variance across the samples in each region for each gene. Because higher variability usually exists for higher expressed genes, instead of using a unified variance value for all genes we modeled the relationship between mean and variance based on a linear model described by Equation 3:

$$\log_2 \sigma_{ij}^2 = \alpha + \beta \log_2 \mu_{ij} + \varepsilon \tag{3}$$

where σ_{ij}^2 is the variance for gene j in region i. The estimated variance $\hat{\sigma}_{jk}^2$ for gene j in mixed sample k was obtained using the linear fit coefficients (α, β) and the expression signatures X_{jk} as in Equation 4. Finally, the expression value for each mixed sample was simulated with a normal distribution with mean X_{jk} and standard variance $\hat{\sigma}_{jk}^2$ (Equation 5). To consider different levels of overall variability, we employed a multiplicity parameter π to change the magnitude of the variance. The value of $\pi = 1$ corresponds to the observed variance in the brain data. Negative values generated based on Equation 5 were replaced by zeros.

$$\log_2 \hat{\sigma}_{jk}^2 = \alpha + \beta \log_2 X_{jk} \tag{4}$$

$$\mathrm{exp}_{jk} \sim \mathrm{Norm}\left(X_{jk}, \left(\pi \hat{\sigma}_{jk}\right)^2\right) \tag{5}$$

Deconvolution was performed with the number of mixed samples ranging from 4 to 25 and the magnitude of the standard deviation multiplier ranging from $\pi = 0$ to 3 with 0.5 intervals. Each run was performed with five replicates.

Supplementary Results

Comparison of the performance of CellDistinguisher and CAM on three independent datasets

For every dataset, the distinguisher gene sets for the different cell types were computed with CellDistinguisher using the default parameters. The sample compositions were computed with the ssKL algorithm implemented in the CellMix package [6] using the top 100 distinguishers for each cell type.

Dataset GSE11058

The dataset consists of 24 samples, each having microarray expression data for either one of four single human immune cell lines or mixtures of various relative proportions [7]. Measurements were performed in triplicates. To objectively judge the performance of the deconvolution tools, it is important to mention that the average coefficients of variation across the experimentally measured expression values of three sample replicates were in the range of 24.9-30.7%. The coefficients of variations were computed across the replicates, followed by averaging over all genes within a sample type. The maximum difference in the predicted sample composition based on the CellDistinguisher signatures relative to the experimental composition was 22.8% when using the individual values, with an average difference of 5.4%. Using averages across samples resulted in very similar accuracy with a maximum composition difference of 21.6% and an average of 5.8%.

This dataset has expression values for 54,675 probes. After extensive filtering to remove the very high and low values, the CAM analysis was run on approximately 13,000 probe sets [2]. For the CellDistinguisher analysis, we used the default process, which included removing outliers defined as the probes with the top 0.1% expression values and those for which the maximum expression value was more than 3 times higher than the next highest expression value for that probe. This filtering removed a only few hundred cells, leaving approximately 54,000 probes for the analysis.

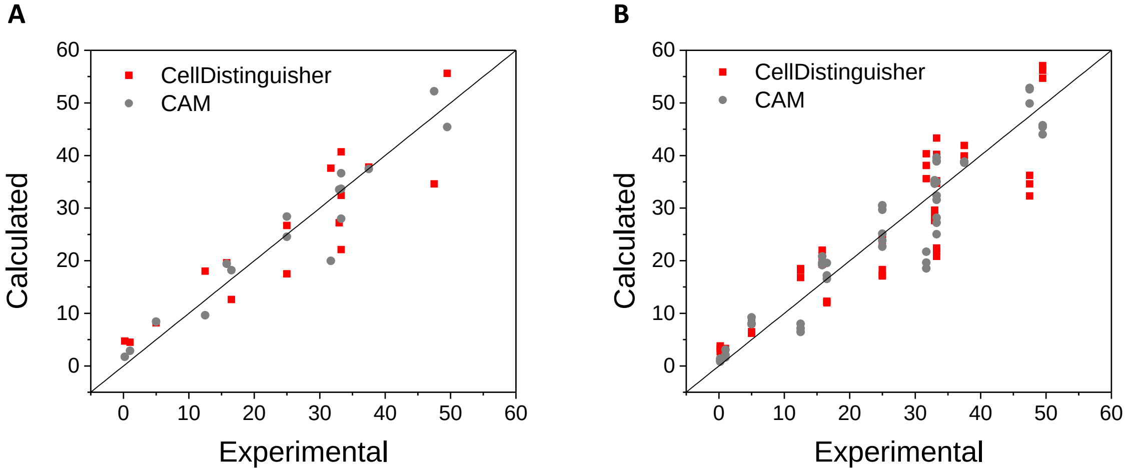

Figure S1. Comparing the calculated and experimental compositions of the mixed samples. Panel A: The calculated values were obtained after averaging the expression values across the sample triplicates. Input to the algorithms were the average expression values in 4 pure and 4 mixed samples. The CAM values were taken from the Supplementary Table 2a of Wang et al [2]. Panel B: The calculated and experimental values of the mixed samples, each replicate handled as an individual sample. Input to the algorithms were 12 pure and 12 mixed samples. The CAM values were taken from the Supplementary Table 2b of Wang et al [2].

Table S1. Calculated and expected compositions of samples. The analyses were run on averages expression values computed across the three replicates of each sample type. Calculated CAM compositions were only provided for the mixed samples (Wang et al, Suppl. Table 2a)[2].

SampleName	Component	CellDistinguisher	Expected	CAM
Jurkat_avg	Jurkat	91.2	100	
IM.9_avg	Jurkat	2.4	0	
Raji_avg	Jurkat	3.1	0	
THP.1_avg	Jurkat	8.2	0	
MixA_avg	Jurkat	26.7	25	28.39
MixB_avg	Jurkat	8.2	5	8.4
MixC_avg	Jurkat	4.5	1	2.85
MixD_avg	Jurkat	4.7	0.2	1.73
Jurkat_avg	IM-9	5	0	
IM.9_avg	IM-9	90.6	100	
Raji_avg	IM-9	6.1	0	
THP.1_avg	IM-9	10.2	0	
MixA_avg	IM-9	18	12.5	9.62
MixB_avg	IM-9	37.6	31.7	19.95
MixC_avg	IM-9	55.6	49.5	45.42
MixD_avg	IM-9	40.7	33.3	27.99
Jurkat_avg	Raji	2	0	
IM.9_avg	Raji	6.3	0	
Raji_avg	Raji	89.5	100	
THP.1_avg	Raji	3.2	0	
MixA_avg	Raji	17.5	25	24.55
MixB_avg	Raji	34.6	47.5	52.22
MixC_avg	Raji	12.6	16.5	18.21
MixD_avg	Raji	22.1	33.3	33.66
Jurkat_avg	THP-1	1.8	0	
IM.9_avg	THP-1	0.7	0	
Raji_avg	THP-1	1.4	0	
THP.1_avg	THP-1	78.4	100	
MixA_avg	THP-1	37.8	37.5	37.44
MixB_avg	THP-1	19.6	15.8	19.43
MixC_avg	THP-1	27.2	33	33.52
MixD_avg	THP-1	32.4	33.3	36.62

Table S2. Pearson correlation coefficients between calculated and expected compositions of the mixed samples using average expressions across replicates or individual values.

		CellDistinguisher	CAM
Expected	Average of replicates	0.90796	0.96048
Expected	Individual samples	0.90676	0.94857

Table S3. Pearson correlations between the calculated expression values of the signature genes and their average expression values in the pure cell types. CAM values are from Supplementary table 4e of Wang et al [2].

	Jurkat	IM-9	Raji	THP-1
CellDistinguisher	0.9563	0.9006	0.9794	0.9838
CAM	0.9521	0.9758	0.9745	0.9633

Dataset GSE19380

This dataset consists of microarray expression values for 31,099 probes for 26 samples derived from primary neuronal, astrocytic, oligodendrocytic and microglial cultures, as well as from RNA mixtures of these [8]. For the CAM analysis, sample 8 (A4 containing astrocytes) was removed because of suspected poor quality. All other mixtures containing cells from this culture (samples 18, 20, 22, and 24) as well as samples containing microglia were also removed. On the remaining 15 samples, Wang and coworkers reported a correlation coefficient of 0.99 between the estimated and expected subpopulation proportions, while the gene expression profiles were estimated with a correlation coefficient of 0.94-0.99 relative to the ground truth.

The CellDistinguisher analysis on all 26 samples resulted in a Pearson correlation coefficient of 0.98 between predicted and expected sample compositions. On the 15 selected samples, we estimated the subpopulation proportions with an almost perfect correlation coefficient of 0.9954 relative to the expected values (Fig. S2). This is nearly identical to the 0.99 value obtained with CAM.

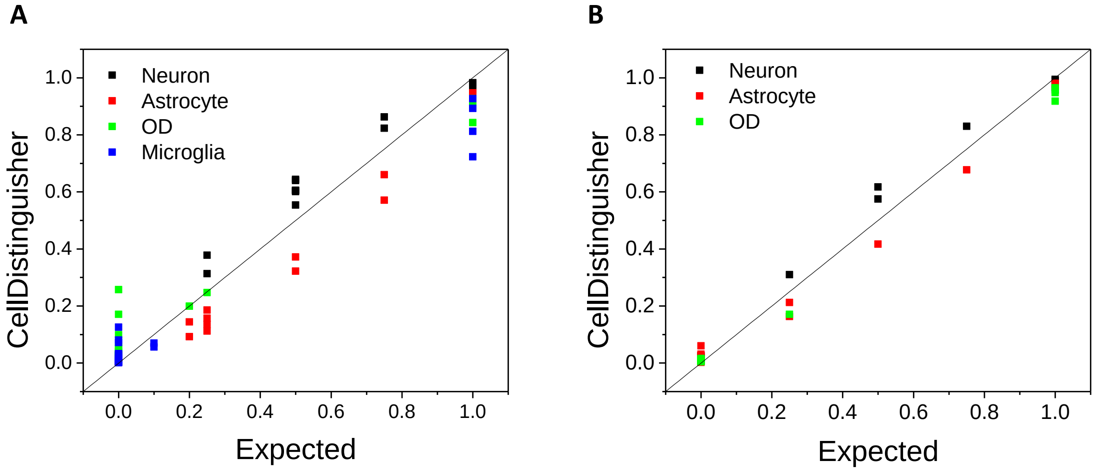

Figure S2. Predicted sample compositions following CellDistinguisher analysis relative to expected values using all 25 samples (Panel A) or just 15 samples used in the CAM analysis (Panel B).

The comparison between the expected and predicted cell type signatures obtained with the dataset of 15 samples and 3 cell types is summarized in Supplementary Table S4. Excellent correlations were obtained between the predicted expression values of the cell type distinguisher genes and their experimentally determined expression values in pure cell cultures. The Person correlation values were 0.904-0.963 for neurons, 0.980-0.984 for astrocytes and 0.839-0.963 for oligodendrocytes. This is especially remarkable given that the correlations between the experimental replicates were lower, especially for oligodendrocytes. These values are very close to the ones reported for CAM (0.94-0.99) with no filtering done other than the default process for removing outlier probes.

Table S4. Predicted cell type names (Neuron, Astrocyte, OD) are highlighted in yellow, while the experimental pure cell lines (N1-O4) are highlighted in green.

	Neuron	Astrocyte	OD	N1	N2	N3	N4	A1	A2	A3	O1	O2	O3	O4
**Neuron**	1	-0.25	-0.24	0.963	0.943	0.904	0.917	-0.24	-0.26	-0.26	-0.22	-0.23	-0.21	-0.22
**Astrocyte**	-0.25	1	-0.22	-0.24	-0.23	-0.22	-0.22	0.98	0.984	0.98	-0.16	-0.21	-0.17	-0.17
**OD**	-0.24	-0.22	1	-0.22	-0.21	-0.21	-0.22	-0.19	-0.22	-0.22	0.917	0.946	0.839	0.963
**N1**	0.963	-0.24	-0.22	1	0.982	0.773	0.79	-0.24	-0.25	-0.26	-0.2	-0.21	-0.2	-0.2
**N2**	0.943	-0.23	-0.21	0.982	1	0.727	0.746	-0.22	-0.24	-0.25	-0.2	-0.21	-0.19	-0.2
**N3**	0.904	-0.22	-0.21	0.773	0.727	1	0.993	-0.21	-0.23	-0.23	-0.2	-0.2	-0.19	-0.2
**N4**	0.917	-0.22	-0.22	0.79	0.746	0.993	1	-0.21	-0.23	-0.23	-0.2	-0.21	-0.19	-0.2
**A1**	-0.24	0.98	-0.19	-0.24	-0.22	-0.21	-0.21	1	0.94	0.931	-0.13	-0.18	-0.16	-0.15
**A2**	-0.26	0.984	-0.22	-0.25	-0.24	-0.23	-0.23	0.94	1	0.995	-0.15	-0.2	-0.17	-0.16
**A3**	-0.26	0.98	-0.22	-0.26	-0.25	-0.23	-0.23	0.931	0.995	1	-0.15	-0.2	-0.17	-0.17
**O1**	-0.22	-0.16	0.917	-0.2	-0.2	-0.2	-0.2	-0.13	-0.15	-0.15	1	0.938	0.585	0.847
**O2**	-0.23	-0.21	0.946	-0.21	-0.21	-0.2	-0.21	-0.18	-0.2	-0.2	0.938	1	0.71	0.856
**O3**	-0.21	-0.17	0.839	-0.2	-0.19	-0.19	-0.19	-0.16	-0.17	-0.17	0.585	0.71	1	0.837
**O4**	-0.22	-0.17	0.963	-0.2	-0.2	-0.2	-0.2	-0.15	-0.16	-0.17	0.847	0.856	0.837	1

Dataset GSE19830

The dataset contains microarray gene expression data obtained from pure and mixed rat tissues (brain, liver, lung), 3 technical replicates each [9]. To compare the CellDistinguisher results to the CAM ones, we computed the average linear expression values across the replicates, then ran the CellDistinguisher analysis on these average values. Only the mixed samples were used for identifying the distinguisher genes. The compositions were determined using both the default deconvolution algorithm of the CellDistinguisher package and ssKL from the CellMix package, with the former producing slightly better results (correlation between calculated and expected compositions of 0.993 vs 0.939). When computed this way, the correlation between the calculated and expected compositions was 0.993 for CellDistinguisher and 0.979 for CAM (Fig. S3). To compute the correlation, the CAM-derived composition values were from Wang et al, Supplementary Table 7 [2]. The correlation between the CellDistinguisher and CAM compositions were 0.995. When the replicates were analyzed as independent samples and the compositions were averaged across the replicates, the correlation for CellDistinguisher versus the expected values was 0.989, almost identical to the one obtained with averaging the expression values before the analysis. Either way, the performance of CellDistinguisher was very similar to that of CAM for this dataset.

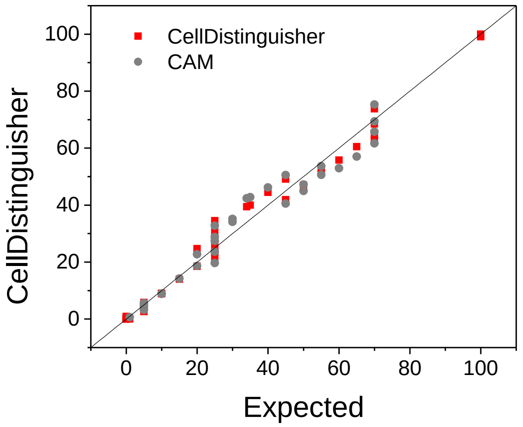

Figure S3. Predicted sample compositions following CellDistinguisher analysis of dataset GSE19830 relative to expected values were compared to those obtained by using the CAM algorithm by Wang et al [2].

References

1. Arora S, Ge R, Halpern Y, Mimno D, Moitra A, Sontag D, et al., editors. A Practical Algorithm for Topic Modeling with Provable Guarantees. Proc 30th International Conference on Machine Learning; 2013; Atlanta, Georgia, USA.
2. Wang N, Hoffman EP, Chen L, Zhang Z, Liu C, Yu G, et al. Mathematical modelling of transcriptional heterogeneity identifies novel markers and subpopulations in complex tissues. Sci Rep. 2016;6:18909. Epub 2016/01/08. doi: 10.1038/srep18909. PubMed PMID: 26739359; PubMed Central PMCID: PMC4703969.
3. Hawrylycz MJ, Lein ES, Guillozet-Bongaarts AL, Shen EH, Ng L, Miller JA, et al. An anatomically comprehensive atlas of the adult human brain transcriptome. Nature. 2012;489(7416):391-9. Epub 2012/09/22. doi: 10.1038/nature11405. PubMed PMID: 22996553; PubMed Central PMCID: PMC4243026.
4. Miller JA, Ding SL, Sunkin SM, Smith KA, Ng L, Szafer A, et al. Transcriptional landscape of the prenatal human brain. Nature. 2014;508(7495):199-206. Epub 2014/04/04. doi: 10.1038/nature13185. PubMed PMID: 24695229; PubMed Central PMCID: PMC4105188.
5. Kang HJ, Kawasawa YI, Cheng F, Zhu Y, Xu X, Li M, et al. Spatio-temporal transcriptome of the human brain. Nature. 2011;478(7370):483-9. Epub 2011/10/28. doi: 10.1038/nature10523. PubMed PMID: 22031440; PubMed Central PMCID: PMC3566780.
6. Gaujoux R, Seoighe C. CellMix: a comprehensive toolbox for gene expression deconvolution. Bioinformatics. 2013;29(17):2211-2. Epub 2013/07/05. doi: 10.1093/bioinformatics/btt351. PubMed PMID: 23825367.
7. Abbas AR, Wolslegel K, Seshasayee D, Modrusan Z, Clark HF. Deconvolution of blood microarray data identifies cellular activation patterns in systemic lupus erythematosus. PLoS One. 2009;4(7):e6098. Epub 2009/07/02. doi: 10.1371/journal.pone.0006098. PubMed PMID: 19568420; PubMed Central PMCID: PMC2699551.
8. Kuhn A, Thu D, Waldvogel HJ, Faull RL, Luthi-Carter R. Population-specific expression analysis (PSEA) reveals molecular changes in diseased brain. Nat Methods. 2011;8(11):945-7. Epub 2011/10/11. doi: 10.1038/nmeth.1710. PubMed PMID: 21983921.
9. Shen-Orr SS, Tibshirani R, Khatri P, Bodian DL, Staedtler F, Perry NM, et al. Cell type-specific gene expression differences in complex tissues. Nat Methods. 2010;7(4):287-9. Epub 2010/03/09. doi: 10.1038/nmeth.1439. PubMed PMID: 20208531; PubMed Central PMCID: PMC3699332.
